# Impairment of the Developing Human Brain in Iron Deficiency: Correlations to Findings in Experimental Animals and Prospects for Early Intervention Therapy

**DOI:** 10.3390/ph12030120

**Published:** 2019-08-14

**Authors:** Veronika Markova, Charlotte Holm, Anja Bisgaard Pinborg, Lars Lykke Thomsen, Torben Moos

**Affiliations:** 1 Department of Obstetrics and Gynaecology, Hvidovre Hospital, Copenhagen University Hospital, 2650 Hvidovre, Denmark; 2 Pharmacosmos A/S, 4300 Holbæk, Denmark; 3 Laboratory of Neurobiology, Department of Health Science and Technology, Aalborg University, 9220 Aalborg, Denmark; 4 Fertility Clinic, Juliane Marie Centre, Rigshospitalet, University of Copenhagen, 2100 Copenhagen, Denmark

**Keywords:** developmental, iron deficiency anemia, neonatal, transferrin receptor, treatment

## Abstract

Due to the necessity of iron for a variety of cellular functions, the developing mammalian organism is vulnerable to iron deficiency, hence causing structural abnormalities and physiological malfunctioning in organs, which are particularly dependent on adequate iron stores, such as the brain. In early embryonic life, iron is already needed for proper development of the brain with the proliferation, migration, and differentiation of neuro-progenitor cells. This is underpinned by the widespread expression of transferrin receptors in the developing brain, which, in later life, is restricted to cells of the blood–brain and blood–cerebrospinal fluid barriers and neuronal cells, hence ensuring a sustained iron supply to the brain, even in the fully developed brain. In embryonic human life, iron deficiency is thought to result in a lower brain weight, with the impaired formation of myelin. Studies of fully developed infants that have experienced iron deficiency during development reveal the chronic and irreversible impairment of cognitive, memory, and motor skills, indicating widespread effects on the human brain. This review highlights the major findings of recent decades on the effects of gestational and lactational iron deficiency on the developing human brain. The findings are correlated to findings of experimental animals ranging from rodents to domestic pigs and non-human primates. The results point towards significant effects of iron deficiency on the developing brain. Evidence would be stronger with more studies addressing the human brain in real-time and the development of blood biomarkers of cerebral disturbance in iron deficiency. Cerebral iron deficiency is expected to be curable with iron substitution therapy, as the brain, privileged by the cerebral vascular transferrin receptor expression, is expected to facilitate iron extraction from the circulation and enable transport further into the brain.

## 1. Introduction

Iron deficiency is the most common type of malnutrition in humans [1,2,3,4]. Iron deficiency occurs due to an inadequate intake, excess loss, or increased need, and gradually leads to insufficient functions of many organs, including the bone marrow, and as a consequence, iron deficiency leads to iron deficiency anemia [5,6,7]. According to the WHO, anemia affects 1.8 billion people worldwide, equivalent to approximately 25% of the world’s population. Among this group, approximately 0.5 billion are women of a reproductive age, and in developing countries, the incidence of anemia is even higher and varies during pregnancy from 35% to 56% for Africa, 37% to 75% in Asia, and 37% to 52% in Latin America.

Women of a reproductive age are particularly at risk for developing iron deficiency due to menstrual bleeding and pregnancy. The prevalence of iron deficiency anemia in pregnant women is high, even in industrialized countries with well-established iron supplementation policies, e.g., in Denmark, where iron deficiency with anemia affects roughly 12% of pregnant women. The state of pregnancy greatly increases the demand of iron used in foetal organogenesis and growth. The pregnant body prioritizes the fetal iron supply over the maternal utilization up until a threshold of the maternal iron stores being adequate, with a maternal plasma ferritin concentration of approximately 14 µg/L [8]. When the maternal iron stores exceed this threshold, the placenta actively transports iron into the fetal circulation to ensure adequate iron supply to the fetus [9].

Iron deficiency can adversely affect brain development in fetuses and infants. Whereas most knowledge on the fetal needs for iron during pregnancy has been obtained within hematological, nephrology, and gastrointestinal disciplines, the impact on the fetal brain of maternal iron deficiency during pregnancy remains quite understudied. A higher turnover of iron in the developing brain [10], in addition to the widespread expression of iron-containing proteins, nonetheless dictate the importance of iron for the developing brain [11,12,13,14]. This is particularly underscored by the profound expression of transferrin receptor 1 by dividing neuroprogenitor cells [15,16] and signifies that cells of the developing brain with respect to their need for iron share a range of common conditions with precursor cells participating in bone marrow erythropoiesis and the formation of duodenal enterocytes of the fully developed organism. This review aims to summarize the current evidence on the significance of iron for the developing brain, how iron deficiency may impair functioning of the central nervous system (CNS) in the human brain and brains of experimental animals, and which therapeutic advances available can prevent damage to the developing CNS.

### 1.1. Transport of Iron into the Brain

The brain acquires iron during life in a privileged manner in that its capillary endothelial cells are the only endothelial cells of the entire body that express transferrin receptors [17]. Iron circulates in blood exclusively bound to transferrin, unless pathological conditions like hemochromatosis occurs, which will result in the presence of low-molecular-weight forms of non-transferrin bound iron. The brain capillary endothelial cells form the blood–brain barrier (BBB) that prevents paracellular, non-specific entry of the iron-containing transferrin into the brain [17]. Rather, the brain capillary endothelium regulates iron transport into the brain via the expression of transferrin receptors [9,13,17]. Iron-transferrin attaches to the transferrin receptor, which results in the formation of endocytic vesicles. Recent studies have also shown that the transferrin receptor of the BBB can bind and take up circulating ferritin [18,19,20,21]. These vesicles are slightly acidic, and the lower pH reduces the binding affinity of iron to transferrin, which loosens their binding. The iron, present on its ferric form, is reduced to ferrous iron, which can be transported out of the endosome and into the cytosol by divalent metal transporter 1 (DMT1) [22]. The release of iron from transferrin within the endosome causes the iron-free apo-transferrin to detach from the transferrin receptor, which allows unbound apo-transferrin to recycle to the luminal surface [17].

Unbound ferrous iron is a potent pro-oxidative molecule that needs immediate oxidation [23,24]. Consequently, ferrous iron is either oxidized within the cytosol by ferrous oxidases, e.g., ceruloplasmin, or gets transported into the brain’s extracellular space via the efflux transporter ferroportin, while undergoing oxidation during passage of the cellular membrane [23]. The iron transported across the brain endothelium accordingly occurs in a non-transferrin bound iron form and thereby is a candidate for binding to transferrin present within the brain extracellular space [17].

Iron in the cytosol participates in essential metabolic processes, e.g., participation in mitochondrial respiration via incorporation mitochondrial enzymes. Many cell types of the body also store residual iron as ferritin-iron, as ferritin also has pro-oxidant activity and is capable of oxidizing ferrous iron to store around 4500 iron atoms [25,26]. Of note, however, is that brain capillary endothelial cells hardly express ferritin, except for during development [27], suggesting that virtually all iron present within the brain capillary endothelial cells is immediately directed toward transport across the BBB to ensure its function further inside the brain.

Iron is also transferred to the brain via transfer across choroid plexus epithelial cells that form the blood–cerebrospinal fluid (CSF) barrier [17]. Like the endothelial cells forming the BBB, the epithelial cells of the choroid plexus also express transferrin receptors [24], but the quantitative relevance of the choroid plexus for iron transport into the brain is of less significance due to their much lower surface than that of brain endothelial cells of the BBB. The choroid plexus nonetheless very likely makes an important contribution to cerebral iron homeostasis, as transferrin of the blood plasma is filtered through the blood–CSF barrier and enters the brain ventricles, while transferrin in parallel is also synthesized and secreted from the choroid plexus to enter the brain ventricles [24]. In sum, this suggests that transferrin of the brain ventricle, and likely also elsewhere in the brain’s extracellular space, is derived from the choroid plexus. In the extracellular compartment of the brain, transferrin is needed to capture non-transferrin-bound iron transported across the BBB or released from neurons and glia. The need for transferrin in the brain’s extracellular space is further underscored by the presence of transferrin receptors on neurons [28]. Surprisingly, transferrin receptors and DMT1 are hardly detected on major glial cells like astrocytes, oligodendrocytes, and microglia [23,28], which suggests that iron enters glial cells as non-transferrin-bound iron, possibly via specific transporters like ZIP14 [29].

### 1.2. Transport of Iron into the Developing and Iron-Deficient Brain

The uptake of iron-containing transferrin at the BBB and blood–CSF barriers and the further transportation of iron into the brain are dramatically upregulated in the developing brain [30,31,32]. The higher iron uptake strongly correlates to a higher expression of transferrin receptors by brain endothelial cells in the developing brain, as evidenced from studies on the rodent brain [30,31]. The upregulated iron transport is attributable to a generally higher need for iron as the progenitor cells of the brain proliferate and differentiate into their final phenotypes [15,16,32,33]. Interestingly, as the cerebral turnover of iron is extremely low and ceases with increasing age, virtually all the iron transported across the brain barriers during development is believed to remain within the brain [10,11,12].

Correspondingly, when the events take place during development, the brain also adapts to conditions with deprivation in iron accessibility by the upregulation of transferrin receptors [28]. When cerebral iron deficiency occurs, the brain profoundly increases the internalization of transferrin receptors in the capillary endothelial cells. The brain also increases the expression of transferrin receptors in neurons in iron deficiency, whereas glial cells, even in stages with robust iron deficiency, fail to express transferrin receptors [28].

Combining iron deficiency with stages of development produces the maximal demand for the brain to mobilize transferrin receptors, but in this context, it is of note that there seems to be an upper limit for the extent to which the brain can adapt. Supporting this notion, the brain failed to increase the expression of transferrin receptors when iron deficiency was subjected to experimental animals during development [31,34]. Therefore, the failure to further increase transferrin receptor expression suggests that the developing brain is particularly vulnerable to severe iron deficiency.

### 1.3. The Significance of Iron for Precursor Cells of the Developing Brain

The availability of iron for the brains’ cells must be adequate to undertake several iron-dependent processes, not only to ensure important functions such as cellular division and differentiation, but also the development of the entire brain [9,11,12], e.g., (i) the complex cellular architecture consisting of neuronal axons ensheathed with myelin synthetized by oligodendrocytes, (ii) the complicated brain–barrier interface supported by astrocytes and pericytes to regulate transport in and out of the brain, and (iii) the establishment of an innate immune system in the brain via the formation of microglia.

The significance of iron for maintaining cellular functions has been covered in former reviews [11,12,35,36,37]. Iron denotes an essential part as the co-factor of several proteins that can be organized into four groups: Non-enzymatic iron-containing proteins; enzymes that use iron-sulfur as a co-factor; enzymes with an iron-containing heme group; iron-containing enzymes without heme or an iron-containing sulfur group. Together, these four groups of proteins undertake essential iron-dependent cellular events, i.e., electron transfer in the mitochondria, regulation of the expression levels of several genes, regulation of cellular division and differentiation, the binding and transport of oxygen, the synthesis of neurotransmitters (in particular serotonin, norepinephrine, and dopamine), the packaging of neurotransmitters in the axon terminal, the reuptake and degradation of neurotransmitters, and the co-factor function for peroxide- and nitrous oxide-generating enzymes for the functioning of immune cells and the intracellular killing of pathogens [11,12,35,36,37]. More specifically, for the developing brain, these above-mentioned cellular iron-dependent processes make their contribution to adequately ensure series of important events ranging from early formation of the neuronal tube to later differentiation of neuronal precursor cells into neurons and glial cells. Iron is very important for the formation of the neuronal tube, of which the formation is abrogated during a conditional lack of transferrin receptors [15,16]. Severe iron deficiency early in life is also expected to impair the forming brain due to the loss of function of the iron-containing enzyme ribonucleotide reductase that is essential for cellular division [38]. This leads to the major concern that remains unexploited, which predicts that developmental iron deficiency during early gestation can cause a permanent reduction in the number of neuronal and glial cells in spite of iron being supplied later in life, e.g., by admitting iron to the neonate [39]. Concerning glial cells, their formation in the developing brain depends on iron-containing enzymes to ensure cellular division and differentiation. Regarding oligodendrocytes, the lack of iron availability during development is thought to significantly affect their capability to form myelin [11,12,40], and current hypotheses concern whether the formation of myelin is permanently affected, even if the iron supply is restored later in life, hence hinting towards a certain time-window during development where iron availability must be adequate to promote myelination [41].

### 1.4. Translational Models of the Brain Development

The effects of iron deficiency on the brain will likely manifest, with the earlier the impacts taking place representing the worst condition. The gestational ages vary dramatically between mammalian species, which is very important to notify when comparing experimental data on the effects of iron deficiency.

**Figure 1:**
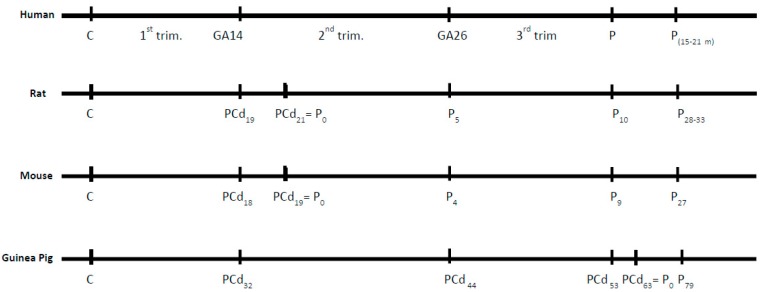
Comparison of the developmental ages of the human, rat, mouse, and guinea pig with respect to myelination of the entire brain. C = day of conception. Psuffixnr = postnatal day. PCd = post-conception day. P = partus. GA = human gestation week corresponding to PCd. trim. = Trimester.

The normal brain development of different mammalian species can be compared (http://www.translatingtime.org/translate) [42]. Events like neurogenesis and myelination in the brains of rats and mice time wise are almost identical (Figure 1), but differences dramatically occur when comparing rodents and humans. Figure 1 also shows that myelination of the human brain compared to myelination in rats and mice is clearly different regarding both timing and the time period of gestation, i.e., myelination of the human brain takes places around post-conception (PC) day 250, which translates to PC day 30 in the rat. The latter is very important to emphasize, because it shows that opposed to the human brain, myelination takes places after birth in the rodent (around P7), which must be taken into account when designing an experimental study on brain development with the purpose of detecting correlations between species.

### 1.5. Evidence of Deleterious Effects of Iron Deficiency on the Developing Brain

The effects of iron deficiency on the gastro-intestinal tract and hematological system are well-described, with the effects being reversible. In contrast, the developing CNS differs from many organs of the body as the impacts are much more prone to be irreversible, even when iron supplies are restored, because the neurons are post-mitotic from the time of birth. The following paragraphs will outline the studies that have been conducted to delineate the effects of iron deficiency on the developing brain, with an emphasis on whether effects were reversible or irreversible. To facilitate the translational relevance, the different species are reported separately based on changes in neuronal and glial functioning, and probable changes in behavior. The selected studies were identified based on a search strategy using PubMed to identify primary research on experimental animal studies and human studies using the following MESH words: iron deficiency, development, brain, or neuro, which revealed approximately 60 relevant studies on experimental animals and observations of the developing human brain during the most recent two decades.

### 1.6. Experimental Animals

The experimental animal data point towards significant effects on the brain during periods of iron deficiency during both the gestational period and after birth (Table 1). The data on small rodents like rats and mice clearly show that the most dramatic effects on the brain development occur when iron deficiency is introduced in pregnancy, whereas data from larger animals like the domestic pig and non-human primates show an influence when iron deficiency is introduced to the offspring (Table 1).

Rodents, especially the laboratory rat, denote the most popular experimental animal for studies of iron deficiency. The gestational period in the rat principally covers the first two trimesters in humans, with the transition between 1st and 2nd trimesters occurring only two days before delivery, whereas the third trimester in the human is reflected in the first weeks after birth in the rat (Figure 1).

The latter points towards a significant difference in the possibilities to compare human and rodent studies, as feeding of this early postnatal rodent no longer occurs via the transfer of nutrients across the placenta, but instead relies on a functioning gastrointestinal system of the neonate. The absorption of iron mainly occurs in the proximal duodenum and is regulated by the iron availability of the duodenal enterocytes. These are mainly under regulation of the general iron status in the neonate via signaling via circulatory levels of hepcidin, which is a hormone synthetized and released from the liver in response to inflammatory stimuli and high circulatory levels of iron [43]. However, inflammation in the neonate may lead to increased levels of hepcidin, as this will negatively affect iron uptake from the gut [6], and hence the rodent as a model of development equal to the third semester in the human fetus represents a model of potential risk. On the other hand, for the study of the effects of iron deficiency during development, the early postnatal rodent represents an accessible model with many possibilities for intervention.

The studies pertained on the developing rodent brain all point towards a deleterious effect of dietary iron deficiency subjected to the mother during pregnancy (Table 1). The effects range from observations based on a direct comparison with normal fed mothers to reports on permanent effects on the brain of the offspring in spite of iron being admitted even early after birth. The effects on the developing rat brain concern structural, biochemical, and behavioral impairments (Table 1). Structurally, influences include structural defects in general brain development [48], and more specifically, the development of dendritic length and arborization, and effects on the formation of synapses [44,53,57,58]. A particular focus in many studies has been the effects of changes in the expression level of genes related to the functioning of synaptic transmission [55,59], vascularization [55], and hormones improving metabolism [61]. Studies have also reported on defects in the synthesis of monoaminergic neurotransmitters [46,47,57], and growth factors [48]. Additionally, studies have reported on behavioral disturbances [44,49,51,52]. A single study has reported on the impaired development of glial cells [65] and the impaired formation of myelin has also been reported [44,63,65], suggesting that earlier studies demonstrating that changes in the profiles of fatty acids in phospholipids are present in iron deficiency relate back to the functioning of the developing oligodendrocytes [11,12,13,40].

The significance of dietary iron deficiency on the developing mouse has gained less attention than that of the rat. Iron deficiency negatively affects the brain weight, iron content, and formation of oligodendrocytes and their myelination [66,80]. As previously mentioned, genetic depletion of the transferrin receptor in the mouse results in severe fetal effects and impaired neurotransmitter formation [15,16]. In the guinea pig, a series of studies have been made on neural transmission in the brain stem, and reportedly deleterious effects of iron deficiency were partly restored by dietary supplementation with polyunsaturated fatty acids, indicating a beneficial effect on otherwise impaired myelination [72,73,74].

Most studies of larger animals have been conducted in postnatal animals, which, for practical reasons, make this approach durable [8]. Studies of gestational iron deficiency performed in the domestic pig report on impaired myelination, but without cognitive effects [72]. Impaired myelination was also reported in piglets, who were only subjected to postnatal iron deficiency [75]. Interestingly, epigenetic regulation is also affected when iron deficiency is present in the piglet brain [74]. Another intriguing study using MRI reports on permanent changes in the brain of the domestic pig in spite of the reversal of brain iron with dietary treatment [73].

In terms of the non-human primate brain, a single study has reported on cognitive effects following gestational iron deficiency, but the effects were not consistent and were largely dependent on the induced dietary regimen [79]. Studies on iron deficiency induced in the offspring have demonstrated that this led to significant changes in nuclear magnetic resonance (NMR)-detectable metabolites and proteomic profiles in CSF, clearly hinting towards impaired cerebral metabolism [76,77]. Another study has concluded that iron deficiency subjected to the offspring led to behavioral deficits that were compensable, suggesting that the effects of iron deficiency were less deteriorating [78].

### 1.7. The Developing Human Brain

In humans, the brain forms very early, and maternal iron deficiency is likely to impair the developing brain during the entire period of pregnancy. Compiling the studies reporting the negative impact of iron deficiency on the formation of the developing brain in experimental animals, the following factors stand out as being particularly important: The timing and the severity of the iron deficiency regimens. These factors are also very important to keep in mind when considering the impact of iron deficiency or iron deficiency with anaemia in the human brain, as they are likely to be the most determinant concerning whether damage is at risk of being irreversible [81,82]. The effects of iron deficiency on brain development were suggested to include the genesis of dendrites and synapses, hence clearly addressing the effects of iron deficiency on differentiation during formation of the human brain, and specifically suggesting an impact on particular brain regions such as the cerebral cortex (i.e., frontal cortex, prefrontal-striatal network, auditive cortex), hippocampus, and striatum [3,82]. Prior studies were clearly limited in access to measurements on brain functioning in real-time and merely relied on correlations between the iron status measured in blood and putative changes in behavior. Infants with low cord-blood s-ferritin and haemoglobin were prone to negative emotions, and they were less alert and difficult to sooth, and in a 5-year follow-up, the children had poorer behaviour and development outcomes, trouble with auditory language skills, and fine motor skills [82,83,84]. A single trial showed that maternal anaemia in pregnancy could be linked to 14% of cases of mental retardation at a 7-year follow-up. It should, however, also be kept in mind that iron deficiency in humans is not likely to be as extreme as can be instituted in experimental animals, and this should indeed be kept in mind when translating data from animal models to hypotheses in human physiology. A valid indicator of the severity in humans is seen when iron deficiency is complicated with anemia. In this situation, the iron transport to the fetus will be prioritized over the maternal iron need unless a certain threshold (ferritin ≈ 14 µg/L) is met [8,83]. Furthermore, in severe cases of iron deficiency with anemia, fetal erythropoiesis is more highly prioritized than neurodevelopment [33,58].

The data obtained from several studies in humans all congregate towards the conclusion that there are significant effects on brain development, both pre-and postnatally (Table 2). Compared to the more extreme situations that invariably relate to the experimental animals, the impairment in iron statuses is not so dramatic in the human brain, and hence also the reported results [83,84,85,86,87,88,89,90,91,92,93,94,95,96,97,98,99,100,101,102,103,104,105,106]. The studies of humans have mainly involved structural analyses on brain growth in real-time [83,84,85,86,87,88,89], neurophysiological measurements of basic functions related to myelination and cranial nerve development [90,91,92,93,94,95,96], and neuropsychological tests of cognition, memory, and personal traits [97,98,99,100,101,102,103,104,105,106].

Concerning the studies on the development of the brain that took morphological approaches, reports indicate that brain volumes, neurogenesis, and iron content are reduced [83,84,85]. This also led to permanent defects in neuronal connectivity years after developmental iron deficiency was recovered [91], which was further associated with a higher risk for psychiatric disorders [87,88], but not for causing autism [89].

Influences of impaired sensory function and neuronal development were also attributed to developmental iron deficiency using auditory brainstem responses (ABR) [90,91,92,93]. However, at least one study brought the reliability of this measure into question [90] and hinted that changes in ABR can have other explanation. Other studies taking a neurophysiological approach showed impairment in the visual input and general brain activity [94,95,96], suggesting that, e.g., dopaminergic neurotransmission is affected [95].

Examining higher functional tasks, the impact on structures and their function in the forebrain have been reported in many studies of the hippocampus (various memory tasks) [97,98,99] and cerebral cortex (social behavior, cognition, association with ADHD) [100,102,103,105,106]. Conversely, behavior was reportedly not affected in two other studies [101,102], which suggest that effects on the developing brain could be subtle unless dramatic maternal iron deficiency occurs.

## 2. Conclusions

The human brain develops throughout the gestational period, ranging from the formation and proliferation of neuroprogenitor cells, to their later migration, and later differentiation into fully developed neurons and glial cells. Severe iron deficiency can negatively impact cell division, neurotic outgrowth and formation of the neuronal network, and myelination in glial cells. Experimental studies in animals, especially the laboratory rat, clearly support that these cellular events can be impacted by developmental iron deficiency. In the human brain, where events in the third semester are reflected in the initial postnatal weeks, reports also point towards the negative impact of iron deficiency during development. The quality of the identified studies reported here, including the number of involved subjects, appears valid, but some limitations subtract the possibilities for overall conclusions. The translational value of the result of the experimental animal is high, but more data obtained in higher animals with a longer gestation than the rodent brain would be appreciated. Concerning the human data, a certain shortage in the number of available studies prevails and more studies monitoring the cerebral function postnatally are needed. With respect to the validity of the results, it must also be emphasized that publication bias may exist towards the demonstration of effects of iron deficiency on brain development. This would leave out negative results that may remain unpublished, and this may play an important role as scientific results on the developing human brain are rather scarce. Investigations on the brain in the gestational period are obviously very complicated, so research on biomarkers from the umbilical cord or chorion villus biopsy would be highly appreciated.

In terms of the prevention of iron deficiency, strategies have not yet been developed to specifically address the developing brain. Supplementation with oral or parenteral iron is possible in pregnancy and postnatally [2], and strategies to halt iron deficiency anemia will likely also improve cerebral iron deficiency as the brain is able to extract iron from the blood due to the expression of transferrin receptors on brain capillaries [39]. Parenteral iron supplementation is being assayed in pregnant women and women with post-partum hemorrhage to generally improve their iron status [2,4,107,108], and this will likely also improve the cerebral iron status.

## Figures and Tables

**Table 1:** Studies in experimental animals showing cerebral effects of iron deficiency (ID) subjected to pregnant females or their offspring. References are listed chronologically after species rather than after specific topics, as the many studies addressed more than a single objective. Most data were obtained from studies on rats. Abbreviations: ABR, auditory brainstem responses; DPOAE, distortion product of otoacoustic emissions; IHC, immunohistochemistry; PUFA, long-chain polyunsaturated fatty acids.

Species	Study Design	Method	Offspring Age	Conclusion	Reference
Rat	ID before conception + Gestational ID	Electrophysiological recordings	P15–P30, P65	Late term effects on synapses in hippocampus in spite of cerebral iron repletion	[44]
	ID before conception	Behavior	P10–Adult	Some persistent effects in spite of iron repletion	[45]
	ID before conception + Gestational ID	Brain iron Neurotransmitters	P35	Behavioral impairments related to persistent loss in dopamine in spite of brain iron reversal	[46]
	Gestational ID + Lactational ID	mRNA	P6–P21	ID from E15 leads to alteration in tyrosine hydroxylase and reversibility in behavior	[47]
	Gestational ID + Lactational ID	mRNA, proteins morphology	P7–P15; P30	Lower BDNF, impaired neuronal differentiation	[48]
	ID before conception + Gestational ID	Behavior	Adult	Detrimental effects of behavioral tasks, sex dependency	[49]
	Gestational ID	Myelination	P25	Impaired myelination with correlation to impairment	[50]
	Gestational ID	mRNA, proteins	P32–P69	Effect of behavior, no effects on motor skills in hippocampus	[51]
	Gestational ID + Lactational ID	Behavior	P65	Permanent changes in behavioral tasks	[52]
	Gestational ID + Lactational ID	mRNA morphology	P7–P65	Permanent changes in mRNA of neuronal markers and dendritic branching in spite of postnatal reversal to normal diet	[53]
	Gestational ID	mRNA, T3, T4	P12	Marked reduction in T3, T4	[54]
Rat	ID before conception + Gestational ID	ABR, DPOAE	P0–P45	First trimester displays profound changes in auditory brain stem response	[55]
	Gestational ID + Lactational ID	MRI, NMR	P7–56	Restoration of brain iron, permanent size reduction in hippocampus and neurochemical hall-markers in spite of postnatal reversal to normal diet	[56]
	Gestational ID	mRNA	P7–P56	Impaired formation of neuronal network and impaired neuronal plasticity in spite of postnatal reversal to normal diet	[57]
	Gestational ID	Morphology	P21–P40	25% reduction in dendritic length 20% reduction in axonal diameter	[58]
	ID before conception + Gestational ID	ABR	P40	Increased ABR latencies in ID depending on stage of ID	[59]
	Gestational ID + Lactational ID	mRNA	P10–P15	Elevated angiogenic/vasculogenic signaling with increased blood vessel complexity	[60]
	ID before conception + gestational ID	mRNA, T3, T4	E13–P10	Marked reduction in T3, T4 Lowering of thyroid hormone responsive genes	[61]
	Embryonic brain	mRNA	Not available (Cultures at E16)	DFO-induced ID lowers expression of series of markers of dendritic and synaptic development, and mitochondrial function	[62]
	Gestational ID	Tactile stimuli	P1–P32	Tactile stimuli reverse defect myelination and alteration in oligodendrocytes and microglia, but not astrocytes	[63]
Rat	Gestational ID	Pro/anti-oxidant	P0–P70	Age- and iron-dependent levels of oxidative stress profiling	[64]
	Gestational ID	mRNA, IHC	P21, P35	Defect myelination, alteration in glial cells	[65]
Mouse	Gestational ID Brain iron	Hematology	E17–E18	Effect of brain weight, lower brain iron	[66]
	ID in offspring	Brain iron	Adult	Correction of cerebral ID with parenteral iron	[67]
Guinea Pig	Gestational ID + Lactational ID	ABR	P9–P24	Effect of ABR in ID Part restoration with PUFA treatment	[68]
	Gestational ID + Lactational ID	ABR	P24	Effect of ABR in ID	[69,70]
Domestic Pig	Gestational ID + Lactational ID	Cognitive tasks	0–4 weeks after birth	No cognitive deficits	[71]
	ID in offspring	MRI	0–6 weeks after birth	Cerebral ID, alteration in brain tissue composition persists in spite of iron repletion	[72]
	Lactational ID	RNA analysis	4 weeks after birth	Change in hippocampal DNA methylation and gene regulation	[73]
	Gestational ID	MRI, IHC	PD 2–30	ID after PD 14 detriments white matter	[74]
Monkey	ID in offspring	1H NMR	Infancy	Change in metabolomic profile in CSF	[75]
	ID in offspring	Proteomic	Infancy	Persistent change in proteomic profile in CSF	[76]
	ID in offspring	1H NMR	Infancy	Metabolomic profile in CSF predicts effects of ID on brain iron metabolism	[77]
	ID in offspring	Cognitive tasks	Infancy	Only initial cognitive + behavioral deficits	[78]
	Gestational ID + Lactational ID	Cognitive tasks	Infancy	Cognitive and emotional effects present, but vary with protocol	[79]

**Table 2:** Studies of humans showing cerebral effects of iron deficiency (ID) or iron deficiency with anemia (IDA) subjected to pregnant females or their offspring. References are listed after specific topics. Abbreviations: ABR, auditory brainstem responses; LBW, low birth weight; No. F/O, numbers of patients (females/offspring); PND, postnatal day; VEP, visually evoked potentials.

Study Objective	Evidence of ID	Infant Age	No. F/O	Conclusion	Reference
Fetal brain development					
Normal development	Maternal IDA	PND 3–5 days	/70	Maternal IDA adversely affects l hippocampal morphogenesis and fetal production of BDNF	[83]
Normal development	Maternal IDA	18 months	331/	Maternal ID at 34 weeks associated with lower motor scores	[84]
Normal development	Normal iron status	7–11 years	/39	MRI iron content in basal ganglia influences spatial intelligence	[85]
Brain connectivity	Infant IDA	Mean 21.5 years	/31	Different patterns of functional connectivity between former IDA and control young adults	[86]
Risk of schizophrenia	Maternal IDA	Prospective study	/6872	Maternal ID as risk factor for schizophrenia in offspring	[87]
Cerebral functions	IDA in adults	Adult	/2957	IDA associated with increases in psychiatric disorders	[88]
Autism	Infant IDA	2–7 years	/102	No evidence between IDA and autism	[89]
**Basic cerebral functions**					
ABR	LBW	PND 42–6 months	/285	Iron supplements did not improve ABR, but ABR was discarded as measure of impairment in ID	[90]
ABR	Maternal IDA	PND 2, 3 months		ABR closely related to severity of maternal and neonatal iron status	[91]
ABR	Infant IDA	6–24 months		Prolonged latencies in ABR traces in IDA	[92]
ABR	Infant IDA	<48 h	/90	Latent iron deficiency associated with abnormal ABR	[93]
VEP	Infant IDA	6–24 months	/50	Negative correlation between severity of IDA and VEP latencies	[94]
Eye-blinking rates	Infant IDA	9–10 months	61	Increased eye-blink rats consistent with low dopamine function in IDA	[95]
EEG recordings	Infant IDA	0, 9 months	/80	ID associated with EEG asymmetry	[96]
**Memory**					
Memory	Infant IDA	8–10 years	/201	Iron supplementation substantially restores cognitive capabilities	[97]
Execution, memory	Infant IDA	19 years	/114	Chronic impairment of functions related to frontostriatal-connections (executive functions), and hippocampus (recognition memory)	[98]
Recognition memory	Infant IDA	6–18 months	/209	Sustained effects on memory in 10-year follow-up in spite of oral supplement in early life	[99]
Higher cerebral functions					
Social-emotional behavior	Infant IDA	9–10 years	/77	Social-emotional behavior associated with ID	[100]
Behavior	Normal	6–8 years	/264	Fe supplementation in pregnancy without consistent effect on behavior	[101]
Cognition	Infant IDA	1–3 years	/3	Improvement in cognition once iron stores were restored	[102]
Cognition	Infant IDA	Mean age 12.0		Reduced cognitive performance	[103]
Cognition	Infant IDA	12 months	828/828	No effect of IDA on cognition or motor development	[104]
ADHD symptomology, IQ	Infant IDA	2.5–5 years	/123	Effects of early deprivation and ID on ADHD symptoms and IQ years after adoption	[105]
ADHD symptomology	Infant IDA	Mean age 11.0		IDA associated with ADHD	[106]

## Abbreviations

BBB	blood–brain barrier
CNS	central nervous system
CSF	cerebrospinal fluid
